# The effect of *curcumin* ointment on knee pain in older adults with osteoarthritis: a randomized placebo trial

**DOI:** 10.1186/s12906-020-03105-0

**Published:** 2020-10-08

**Authors:** Neda Jamali, Mohsen Adib-Hajbaghery, Alireza Soleimani

**Affiliations:** 1 Trauma Nursing research Center, Faculty of Nursing and Midwifery, Kashan University of Medical Sciences, Kashan, Iran; 2 Internal Medicine Department, Kashan University of Medical Sciences, Kashan, Iran

**Keywords:** *Curcumin*, Ointment, Knee pain, Elderly, Osteoarthritis

## Abstract

**Background:**

Some studies have shown the effect of oral administration of *curcumin* on knee pain. However, limited studies are available on the effect of topical *curcumin.* This study aimed to investigate the effect of *curcumin* ointment on knee pain in older adults with osteoarthritis.

**Methods:**

This double-blind randomized placebo trial was conducted on 72 older adults with knee pain associated with osteoarthritis. The subjects were randomly assigned into an intervention and a placebo group to apply either *curcumin* 5% ointment or Vaseline ointment twice daily for 6 weeks. Using a Visual Analog Scale, the severity of knee pain was measured at the beginning of the study, at the end of the fourth and sixth week. Data were analyzed using descriptive and inferential methods.

**Results:**

The mean baseline knee pain intensity was not significantly different between the two groups (*P* = 0.15). The mean pain intensity was significantly lower in the intervention group than in the placebo group at the third measurement (*P* = 0.02). The repeated-measures analysis showed that over time, the *curcumin* significantly decreased the mean pain intensity in the intervention group (*P* = 0.001). The mixed model showed an absolute difference of 1.133 (i.e. 11.33 mm) score which signifies a medium effect size and that the patient in the intervention group achieved the minimal clinically important difference.

**Conclusion:**

Topical administration of *curcumin* 5% ointment can significantly reduce knee pain in older adults with knee osteoarthritis. *Curcumin* ointment can be used as an alternative treatment in older adults with knee pain associated with osteoarthritis.

**Trial registration:**

Retrospectively registered in the Iranian Registry of Clinical Trials (IRCT) (IRCT20100403003618N6, 2019-03-08), https://en.irct.ir/trial/37155

## Introduction

Knee pain is one of the most common health problems in older adults. Knee osteoarthritis is one of the most important causes of chronic knee pain in older adults [1]. Osteoarthritis has a progressive course. It is one of the leading causes of disability in older adults, causing stiffness, swelling and joint instability, impaired motor function, muscle weakness, imbalance, frequent falls, hospitalization, and dependency [1, 2] and reduces the quality of life of the older adults [3]. The prevalence of osteoarthritis in people aged 65 and over is reported to be between 60 and 90% [2]. A study also reported that about 10% of men and 18% of women over 60 have knee osteoarthritis [4].

In Iranian people, the knee joint is more vulnerable and the prevalence of osteoarthritis and knee pain is higher due to various factors such as the inclination to sit on the ground, the form of the toilets, less participation in sports activities, standing cooking and housekeeping and other lifestyle-related factors [5]. It is reported that 33% of Japanese community-dwelling older adults experience knee pain [6] however, this rate is about 63% in Iran [7].

*A wide range of* pharmacological, surgical and conservative mechanical treatments are being used in the treatment of osteoarthritis [8]. Steroids and nonsteroidal anti-inflammatory drugs (NSAIDs) are among the most commonly used medications in osteoarthritis. However, these drugs have a wide range of cardiovascular, renal, gastrointestinal, and hematological side effects [9]. Moreover, due to their neurological side effects, they increase the rate of falls in older adults [10]. Age-related impairments in liver and kidney functions also increase the risk of experiencing serious drug side effects and interactions among older adults [11]. On the other hand, the costs of medications and dissatisfaction with the outcomes of ordinary treatments drive many older adults to self-treatment with herbs and other traditional remedies or to switch their physician [12, 13]. Hence, treatments such as weight loss, physiotherapy, use of walking aids, pool exercises, lifestyle modifications, as well as complementary and alternative therapies such as herbal remedies have been studied and used to reduce knee pain in older adults [8, 14, 15]. Turmeric is one of the well-known herbs in traditional Iranian medicine. Turmeric is a plant of the ginger family, scientifically known as *Curcuma longa*. Dried rhizomes of this plant are used for nutritional and medicinal purposes and contain the active ingredient *curcumin* (chemically called diferuloylmethane) [16, 17]. *Curcumin* has antioxidant, anticancer, antibacterial antiviral, and anti-inflammatory effects [16]. Its therapeutic benefits have also been shown in gastrointestinal [18], cardiovascular [16], hepatic [19], and skin disorders [20, 21] and metabolic syndrome [22]. It also showed beneficial effects in the management of menstrual problems, chest pain diabetes [23], Alzheimer’s disease, rheumatoid arthritis, and strengthening of the immune system [16]. Topical use of turmeric has also been suggested as anti-inflammatory and analgesic in the management of sprain, osteoarthritis [17] and episiotomy wound [24]. Some studies have also reported the effect of turmeric extract on the improvement of osteoarthritis in animal models [25]. Also, a number of studies have examined the effect of oral *curcumin* on human osteoarthritis. In two studies of *curcumin* capsules, the beneficial effects of *curcumin* on the symptoms of osteoarthritis have been reported [26, 27]. In another study, oral capsules containing a combination of *curcumin* and boswellic acid were effective in reducing the pain associated with osteoarthritis. However, it has not been precisely determined that the analgesic effect was due to *curcumin* or boswellic acid and therefore further studies were recommended [28]. Nieman also used oral capsules containing a combination of glucosamine sulfate, methylsulfonylmethane, white willow bark extract, ginger root concentrate, boswellic acid, turmeric root extract, cayenne, and hyaluronic acid. The compound was effective in alleviating joint pain in community-dwelling adults. However, the researchers could not determine which component of the compound had the greatest effect on pain relief [29]. A systematic review also studied the efficacy of *curcumin* and boswellia in patients with knee osteoarthritis and concluded that both *curcumin* and *boswellia* were more effective than placebo for pain relief in these patients. However, the researchers recommended further studies to produce more reliable evidence for clinical practice recommendations [30]. A recent study also reviewed all studies of dietary supplements (such as curcumin) used to treat osteoarthritis and reported that these supplements provided moderate short-term effects on pain and function in patients with hand, hip, or knee osteoarthritis, although the quality of the evidence was low [31]. Another systematic review of the effects of curcumin on arthritis symptoms has also reported that most studies on the effect of *curcumin* have biases and methodological problems. Therefore, further rigorous studies are needed to evaluate the efficacy of *curcumin* on pain and symptoms of arthritis [4].

Furthermore, with reference to the instability of the *curcumin* and its low absorption in the gastrointestinal system, Nelson et al. have concluded that the results of studies using the oral *curcumin* are unreliable [32]. Referring to the same point, Haroyan et al. have concluded that more studies are needed with different strategies to increase the bioavailability of *curcumin* [28]. On the other hand, most studies of the effect of *curcumin* on knee pain used its oral forms and few studies are available on the effect of its topical forms. Some studies have reported that topical use of *curcumin* results in greater bioavailability [33, 34]. A study reported that topical application of *curcumin* was effective in episiotomy wound healing [24]. Another study has also reported the successful topical application of an ointment containing turmeric and some other herbs for relieving pain and stiffness associated with osteoarthritis of the hand and knee [35]. However, the latter study had a small sample size. As the topical application of NSAIDs is in the first line of treatments of OA and there are not many alternatives, and the problems related to the low bioavailability, low absorption, fast metabolism, and fast systemic elimination of oral *curcumin* [28], and since pain relief is one of the key roles of nurses [36], this study aimed to investigate the effect of *curcumin* ointment on the severity of knee pain in older adults with osteoarthritis. The severity of knee pain in the fourth and sixth weeks after the intervention was considered as the primary and the secondary outcomes of the study. Then, the first and the second hypotheses were: mean pain score in the *curcumin* group would be different from the placebo group at the end of the fourth week; and, mean pain score in the *curcumin* group would be different from the placebo group at the end of the 6 week.

## Methods

### Design and participants

**Fig. 1:**
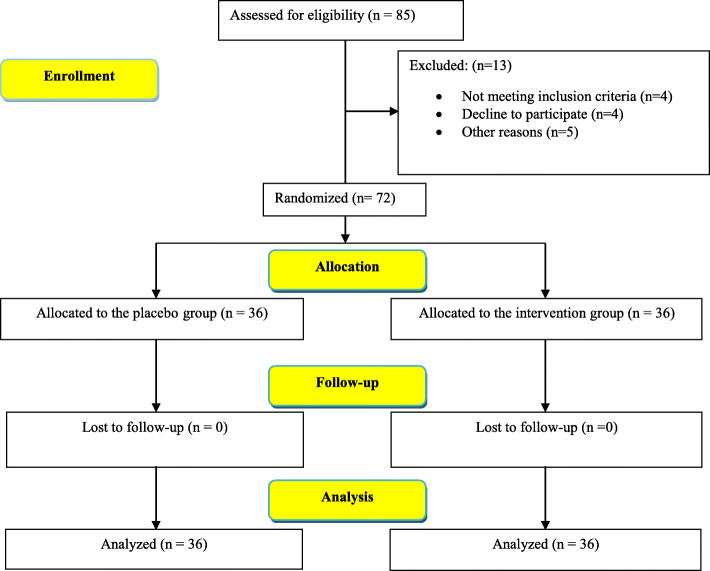
The study flow diagram

This double-blind clinical trial study was performed on 72 community-dwelling older adults with knee osteoarthritis who referred to a physician’s office in Kashan city, Iran. Sampling began on February 20, 2019, and ended on June 22, 2019. The sample size was calculated based on the findings of a former study on the effect of *curcumin* on active rheumatoid arthritis. They assessed pain intensity using a Visual Analog Scale (VAS) of 100 mm and reported that the post-intervention pain score was 27.5 ± 9.4 in the intervention group and 39.2 ± 20.1 in the control group [37]. Then, considering the type I and II errors of 0.05 and 0.2, respectively, and using the following formula $n=\left({\left({Z}_{1-\alpha /2}+{Z}_{1-\beta /2}\right)}^2\times \left({\sigma}_1^2+{\sigma}_2^2\right)\right)/\left.{\left({\mu}_1-{\mu}_2\right)}^2\right)$, the sample size was estimated to be 29 cases for each group. Yet, to compensate for the possible dropouts, 36 eligible older adults were recruited to each group—72 in total (Fig. 1).

Inclusion criteria included an age of 60 and over, no allergy to *curcumin* and plants of the ginger family, lack of cognitive impairment (in examining time, place, and person), full consciousness and ability to complete the study instruments, having a medical diagnosis of knee osteoarthritis, and a willingness to participate in the study. Exclusion criteria included showing a systemic or local allergic reaction to *curcumin* ointment, discontinuation of the ointment before the end of the intervention, or stopping it for at least three consecutive days, a decision to withdraw from the study, death, hospitalization, and moving to another city.

Prior to starting the study, the sampling protocol was formulated using an online block randomization program to assign the supposed participants into 12 blocks of four and a block of two. Thus, eligible older adults who were willing to participate in the study were recruited consecutively and assigned respectively into an intervention or a placebo group based on the blocks structure.

All patients were firstly visited by the physician and he confirmed the knee OA and other eligibility criteria, prescribed them the *curcumin* ointment or placebo according to the blocks structure, gave them a can of the concerned ointment, and then introduced them to the second researcher to complete the study instruments and teach them how to use the prescribed ointment.

### Data collection instruments

Two instruments were used for data collection namely a personal and clinical characteristics questionnaire as well as a visual analog scale (VAS) for assessing the severity of knee pain in the fourth and sixth weeks after the intervention as the primary and the secondary outcomes of the study. The personal and clinical characteristics questionnaire included items on participants’ age, gender, education level, duration of illness, weight (kg), height (cm), body mass index (BM) I, regular physical activity, use of analgesic, sedatives, and hypnotics (yes, no), and if yes, duration and dosage, and also taking non-prescribed over-the-counter medicines and non-pharmacological remedies for pain relief.

**Fig. 2:**
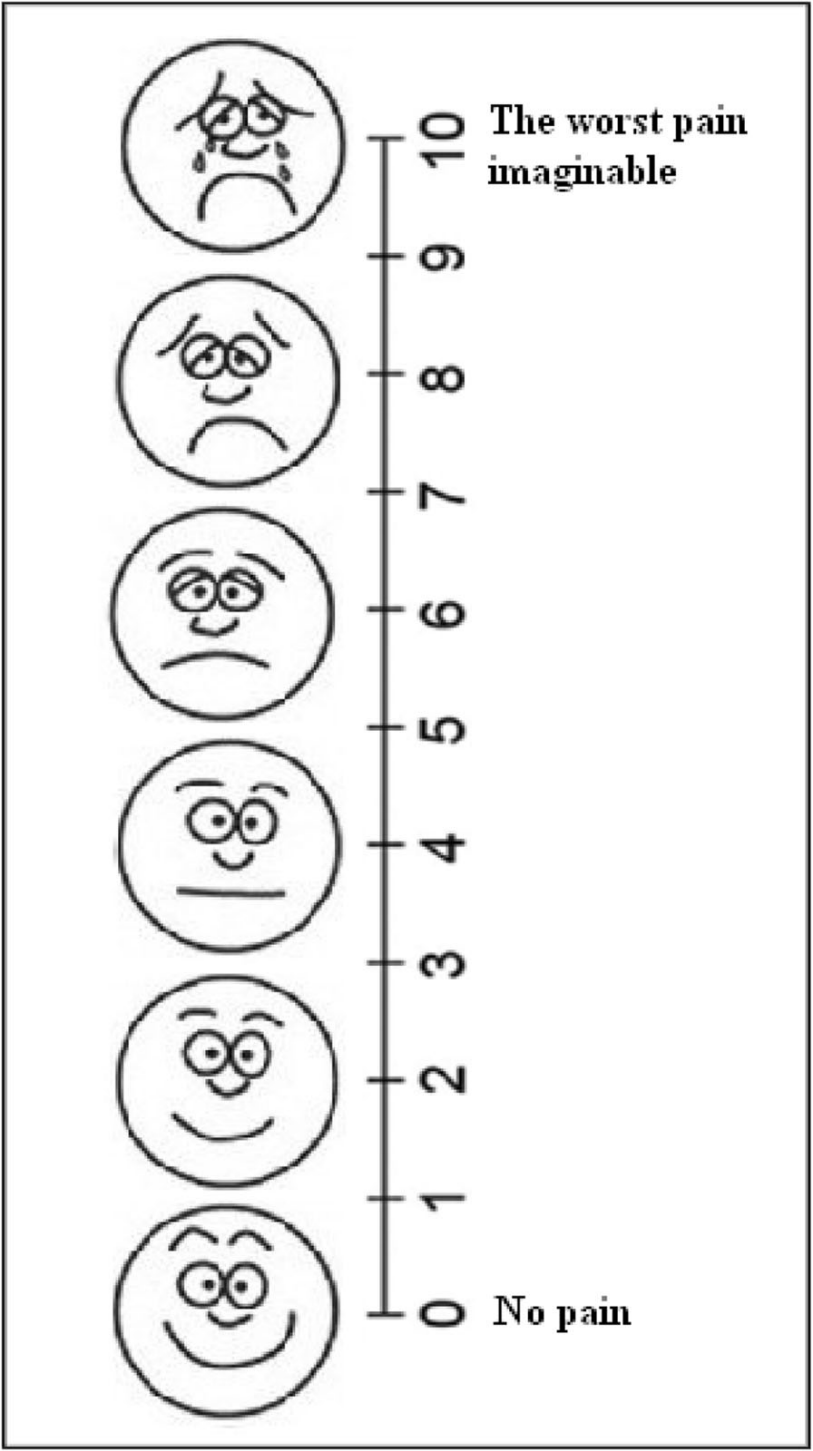
The VAS scale used in the study (english translation)

The second part of the data collection tool included a VAS for measuring the severity of knee pain experienced in the past 24 h. The VAS consisted of a graded 10 cm (i.e. 100 mm) column and the Wong-Baker Faces Pain Rating Scale (WBFPRS). Descriptors were placed at each end of the column (0 = no pain and 10 = the worst pain imaginable). The WBFPRS also consisted of a series of faces ranging from a happy face at 0, or “no pain”, to a crying face at 10, which represents “the worst pain imaginable” (Fig. 2). After explaining the patient on how to use the scale, the researcher showed the scale to every patient and asked him/her to look at the graded column and the faces, and choose a face and a point on the column that best illustrates the knee pain they are experiencing. Then, the researcher marked an X on the column in a place that corresponded to the severity of pain expressed by the patient.

### *Curcumin* and placebo ointments preparation

To produce 5% *curcumin* ointment, a pharmacist mixed 95 g of Vaseline and five grams [24] of dried *curcumin* extract 90% powder (Sample Serial No.: I954212, Batch No.: 908001) purchased from the Barij Essence company, Kashan, Iran.

To keep the study blind form the physician and the patients, *curcumin* and placebo (Vaseline) ointments were prepared in 100 g yellow-colored cans with similar shape, size and weight, and then all cans were coded by the pharmacist as “a” or “b”. The treating physician and also the patients were not aware of codes. Also, the two ointments (i.e. curcumin and Vaseline) did not differ significantly in smell.

### Intervention

After each patient was examined by the physician and accepted to participate in the study, the physician introduced him/her to the second researcher to be interviewed, complete the study instruments, and be practically trained on how to use the ointment. All the patients were individually interviewed and trained by the second researcher in a separate room at the physician’s office. The patients in the intervention group received *curcumin* ointment and the patients in the placebo group received Vaseline ointment. However, both groups received similar educations. Patients were blind about their group and had no contact with each other which enable them to guess the difference.

After completing the study instruments (T_1_) with the help of a trained research assistant, the researcher explained the study process to the patients, took their written informed consent, and then took an initial skin test on their elbow to ensure not having an allergic reaction. To do so, the researcher applied one fingertip of the ointment on the internal surface of the patient’s arm (1 cm × 1 cm). If redness or any other allergic reactions were not observed after 20 min [38] the researcher trained them on how to use. The second researcher taught all patients about the following items and wanted them to do the 1st time at her presence for ensuring both the researcher and the patients of the correct use:

1. Wash the knee and its surrounding area with lukewarm water and dry it with a clean cotton towel.
2. Using a special cup, remove 1.5 ml of the ointment and apply it on and around the knee.
3. Then, wrap around the joint with a clean, non-penetrating cloth to prevent contamination of his/her clothing.
4. Not to apply any other material on the place.

Patients were asked to repeat this work for 6 weeks, twice daily (once in the morning and once at night, before going to bed). A checklist was also provided to the patient to mark it after each use and they were told that additional drugs will be prescribed in the next visits if necessary.

Patients were instructed to report any tenderness, redness, or itching in the area and to refrain from taking any other topical painkillers on their knees during the study. The contact number was taken from each patient and the researcher contacted them weekly to follow the treatment process. The intensity of knee pain was reassessed by the same research assistant and using the VAS at the end of the fourth (T2) and the sixth week (T3) when the patients were referred for follow-up. At every visit, patients were also questioned about any side effects of the treatment (i.e. redness, itching, rashes, etc).

### Ethical considerations

The study protocol was approved by the Ethics Committee and the Institutional Review Board of Kashan University of Medical Sciences, Kashan, Iran (code: IR.KAUMS.NUHEPM.REC.1397.049, grant number: 97151). Moreover, the study was registered in the Iranian Registry of Clinical Trials (code: IRCT20100403003618N6). At the beginning of the study, participants were ensured that their data would be kept confidential and their participation in and withdrawal from the study would be voluntary. Informed consent was obtained from all participants. Although we provided the participants with explanations about the aims and the process of the study, they were blind to the type of ointment they received. Participation in the study charged them no cost and the rights of the participants were protected according to the Declaration of Helsinki.

### Statistical analysis

Data analysis was carried out using the SPSS software v. 16 (SPSSInc.Chicago, Illinois, USA). Descriptive statistics (frequency, percentage, mean, and standard deviation) were calculated. Kolmogorov-Smirnov test was used to examine the normal distribution of the data. Chi-square or Fisher’s exact tests were used to compare the nominal and categorical variables of the two groups. The independent samples t-test was used to compare the mean of quantitative variables of the two groups. Also, the repeated measures analysis of variance (RM ANOVA) was used to compare the variations of the mean knee pain intensity of the two groups through the three subsequent measurement time points. Greenhouse-Geisser estimation was used for epsilon correction and independent-samples *t-*test for pairwise comparisons. Moreover, independent and paired sample t-tests were used for between- and within-group comparison of the mean pain changes during the study. Also, the mixed-effects model was used to examine the effect of baseline knee pain level on post-intervention knee pain. The level of significance was < 0.05.

## Results

Of the 85 patients who were assessed for eligibility, 72 completed the study. Of these, 62.5% were female, 59.7.3% were literate, 55.5% lacked regular physical activity, and 26.4 and 27.8% were taking analgesics and hypnotics during the study. The mean age of the intervention and placebo group was 68.86 ± 6.27 and 67.94 ± 6.72 years, respectively (*P* = 0.80). Also, the two groups did not significantly differ with respect to their mean height, weight, BMI, and other demographic characteristics (*P* <  0.05; Table 1).

**Table 1:** Between-group comparisons of the participants’ demographic and clinical characteristics

Variables	Group		***P*** value
	Placebo, Mean ± SD	Intervention, Mean ± SD	
Age	67.94 ± 6.72	68.86 ± 6.27	0.800^a^
Weight (kg)	75.50 ± 12.44	76.02 ± 12.62	0.560 ^a^
Height (cm)	165.50 ± 6.58	165.63 ± 7.04	0.640 ^a^
BMI	27.54 ± 3.96	27.59 ± 3.43	0.578 ^a^
Disease duration, year	6.91 ± 4.68	7.22 ± 4.46	0.654 ^a^
	N (%)	N (%)	
Gender			
Female	23 (63.9)	22 (61.1)	0.99 ^b^
Male	13 (36.1)	14 (38.9)	
Education level			
Illiterate	13 (36.1)	16 (44.4)	0.34 ^c^
Elementary school	12 (33.3)	12 (33.3)	
Secondary school	4 (11.1)	6 (16.7)	
High school and higher	7 (19.5)	2 (5.6)	
Regular physical activity			
Yes	18 (50.0)	14 (38.9)	0.47 ^b^
No	18 (50.0)	22 (61.1)	
Analgesic use (during the study)			
Yes	8 (22.2)	11 (30.6)	0.59 ^b^
No	28 (77.8)	25 (69.4)	
Name of the analgesic used			
Piroxicam	2 (5.6)	3 (8.3)	0.90 ^c^
Celecoxib	2 (5.6)	5 (13.9)	
Gabapentin	3 (8.3)	2 (5.6)	
Naproxen	1 (2.8)	1 (2.8)	
Use of hypnotics			
Yes	14 (38.9)	6 (16.7)	0.06 ^b^
No	22 (61.1)	30 (83.3)	
Name of the hypnotic used			
Clonazepam	6 (16.7)	3 (8.3)	.99 ^c^
Alprazolam	8 (22.2)	3 (8.3)	

^a:^ Independents samples-test, ^b^: Chi square test, ^c:^ Fisher’s Exact test

As Table 2 shows, the mean baseline (T1) knee pain intensity was not significantly different between the two groups (*P* = 0.111). In the repeated-measures analysis, the Mauchly’s test illustrated that sphericity was not assumed [χ^2^ (2) = 48.55; *p* <  0.001], then the degrees of freedom were corrected using the Greenhouse- Geisser test. The results showed that over time, the *curcumin* significantly decreased the mean pain intensity in the intervention group [F = 119.02, df = 1.329, and *P* = 0.001; Table 2]. However, a significant interaction was observed between time and the mean pain scores in the two groups [F = 40.241, df = 1.329, and P = 0.001]. Considering the observed interaction, the t-test was used to conduct pairwise comparisons between the two groups at T2 and T3. As illustrated in Table 2, the mean pain intensity has significantly decreased in both groups at the end of the fourth week (T2). Although the intervention group experienced a greater reduction in pain, the mean pain scores were not significantly different between the two groups at T2 (*P* = 0.221). The value of between-group difference at T2 was 0.47 (i.e. 4.7 mm in the VAS) which signifies an effect size of 0.291. However, the mean pain intensity was significantly lower in the intervention group than in the placebo group at the end of the sixth week (T3) (*P* = 0.006). The value of between-group difference at this time was 1.14 (i.e. 11.4 mm in the VAS) which signifies an effect size of 0.664.

**Table 2:** The comparisons of the mean knee pain at three measurement time points

Measurement	Group		*P* value (t-test)	RM ANOVA		Mixed model analysis with interaction ^a,b^		
	Intervention, Mean ± SD	Placebo, Mean ± SD		Parameter	*P* value	Parameter	Estimate	*P* value
Baseline pain	6.86 ± 1.60	6.25 ± 1.61	0.111	Time	0.001	Intercept	5.527	.000
Pain after 4 weeks	5.22 ± 1.70	5.69 ± 1.52	0.221	Group	0.001	Baseline pain	0.733	.000
Pain after 6 weeks	4.52 ± 1.78	5.66 ± 1.65	0.006	Group×time	0.001	Time	−0.494	.080
						Intervention group	−1.133	.006
						Baseline pain ×group	−.712	.002
						Baseline pain×time	−0.018	.233

^a.^ Control group is set as the reference group

^b.^ Dependent Variable: Pain

**Fig. 3:**
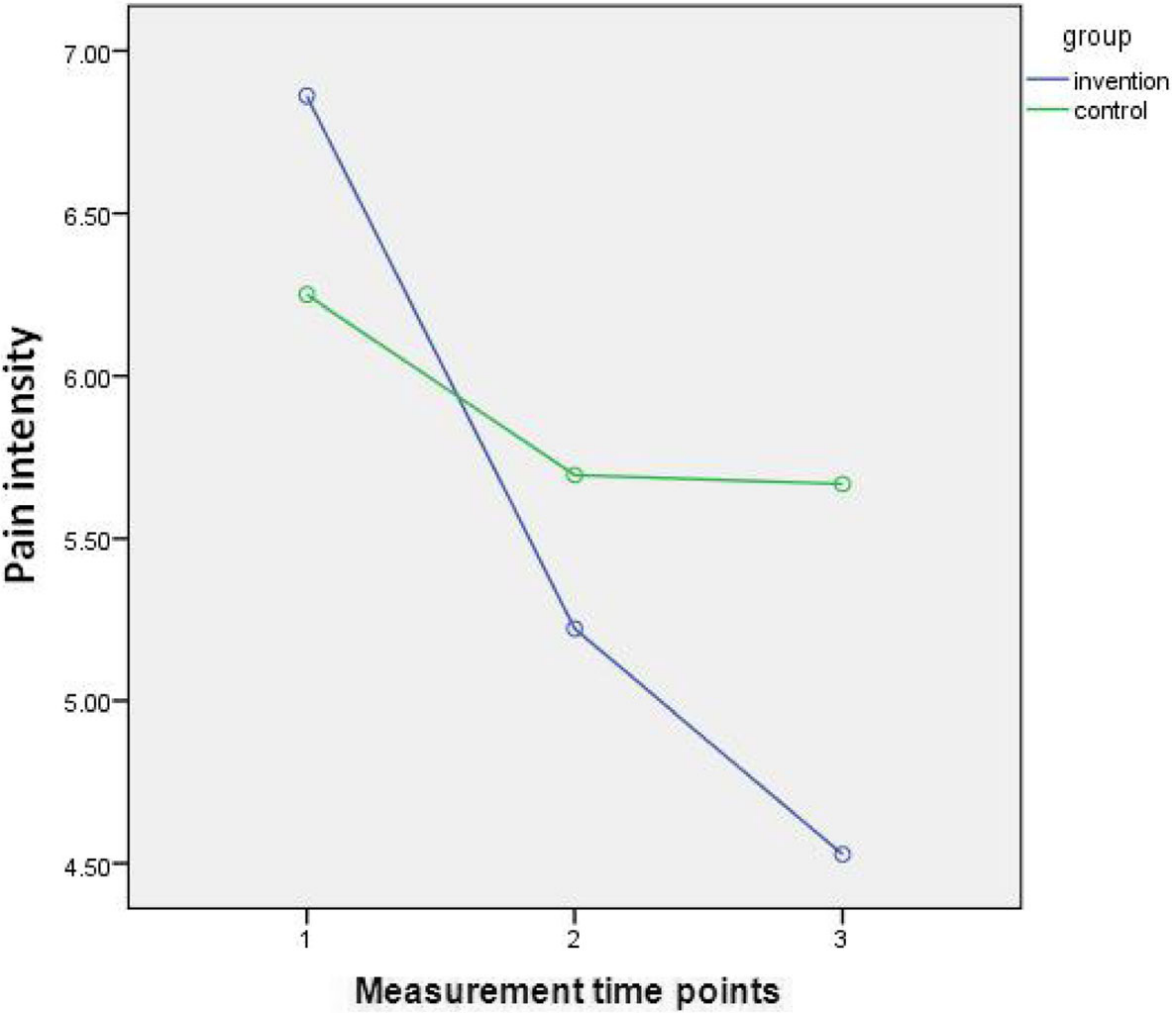
Variations of sleep quality in both groups

Figure 3 shows that the mean pain intensity in the placebo group decreased by 0.56 between T1 and T2 (*P* = 0.71) but it did not significantly change afterward. However, in the intervention group, the amount of pain reduction between T1 and T2 was nearly threefold that of the placebo group (i.e. 1.64 score) (*P* = 0.001) and then continued to decrease at T3. Pairwise comparisons showed that all measurements in the intervention group were significantly different from each other (P = 0.001) and the pain intensity in this group had a significant decreasing trend during the study.

We calculated the changes in pain scores between T1 and T2 and also between T2 and T3. Table 3 shows that between-group differences of changes were statistically significant both for T1-T2 and for T1-T3 (*p* <  0.05). Also, within-group changes were statistically significant in the intervention group.

**Table 3:** Comparison of the mean changes in knee pain scores during the study

Pain change	Group		*P* value ^a^
	Intervention	Placebo	
First (T1-T2)	1.64 ± 0.79	0.56 ± 0.87	< 0.001
Second (T1-T3)	2.33 ± 1.06	0.58 ± 1.05	< 0.001
*P* value ^b^	0.002	0.930	

^a:^ Independents samples-test, ^b^: Paired t-test

According to the linear mixed model effects, both the intervention and baseline pain had significant effects on post-intervention perceived pain (Table 2). The mixed model showed that the overall estimate of pain in the intervention group was 1.133 (i.e. 11.33 mm) which signifies an effect size of 0.63 which is at a medium level, and shows that the intervention group experienced a pain score of 1.133 point less than the placebo group.

During the study, none of the patients reported a local or systemic adverse event due to the use of *curcumin* or Vaseline.

## Discussion

In the present study, the effect of of *curcumin* ointment on the severity of knee pain in older adults with osteoarthritis was investigated. The outcome was assessed at the end of the fourth and sixth weeks. Although the results confirmed the progressive effect of *curcumin* ointment, however, the between-group difference was not statistically significant at the end of the fourth week. The value of the between-group difference was only 0.47 at this time. In other words, patients in the intervention group scored their pain only 0.47 (i.e. 4.7 mm) less than the placebo group which signified a small effect size at the end of the fourth week. Therefore, our first hypothesis cannot be accepted. However, the between-group difference was statistically significant at the end of the sixth week and the value of the between-group difference was 1.14 at this time. In other words, patients in the intervention group scored their knee pain 1.14 (i.e. 11.4 mm) less than the placebo group which signified a medium effect size at the end of the sixth week, and our second hypothesis was accepted. The mixed model also showed an absolute difference of 1.133 (i.e. 11.33 mm) which signifies a medium effect size. In addition to effect size, Polit and Beck (2017) also proposed the standard deviation as a criterion for operationalizing the minimum clinically important difference (MCID) in a clinical trial. This method interprets the results in the light of one-half standard deviation of baseline scores. Accordingly, a change equivalent to one half standard deviation of baseline scores of the comparison group is a good benchmark for interpreting that patients have achieved a minimal clinically important difference [39]. As the baseline standard deviation of the placebo group was 1.61, a change of 0.805 can be a criterion for the improvement in the present study. Then, we can conclude that the absolute difference of 1.133 at the end of the present study shows that the patient in the intervention group achieved a minimal clinically important difference.

Although the placebo group experienced a slight but insignificant decrease in knee pain in the fourth week, this decrease did not continue in the third measurement.

Only a small study of topical application of an ointment containing turmeric and some other herbs in knee and hand arthritis pain is available in which 17 patients used the ointment for 42 days and found it beneficial in the improvement of joint pain and stiffness [35]. A number of studies have also used *curcumin* in older adults with osteoarthritis [26, 28] and reported that it was effective in reducing joint pain and stiffness. However, Nelson et al. reported that *curcumin* is a very unstable compound and its oral administration provides poor bioavailability. This may have led to erroneous results in laboratory and clinical studies. Therefore, further studies are needed to evaluate the effectiveness of its oral extract on joint pain [32]. Referring to the low intestinal absorption, rapid metabolism, low bioavailability, and water insolubility of *curcumin*, Gaffey et al. have also suggested further studies to address its effect on pain due to chronic osteoarthritis [40].

Daily et al. have also reviewed the clinical trials investigating the effect of turmeric extract and *curcumin* on alleviation of arthritis symptoms. They reported that only in half of the 8 articles reviewed did the clinical symptoms improved. In five studies no significant difference was found between the mean pain intensity in the groups used turmeric and those used commercially available pain drugs, and in these studies, only three studies used a proper method for randomization of the subjects [4].

The progressive reduction of knee pain in the intervention group of the present study can be attributed to the local effect of *curcumin*. Turmeric is one of the common herbs in traditional Iranian medicine. In Iranian traditional medicine, a mixture of *curcumin* and egg yolk has been used to reduce pain and accelerate the healing process of sprains, strains, and fractures [41–43] and to reduce joint pain and stiffness [44]. Turmeric extract contains *curcumin* or diferuloylmethane and other volatiles that have antioxidant, antitoxic, anti-free radicals, and anti-inflammatory effects [16]. Some studies have also reported that short-term oral administration of *curcumin* had anti-inflammatory effects similar to hydrocortisone and phenylbutazone [45, 46]. In an animal study, an aqueous extract of *curcumin* showed a significant anti-inflammatory effect [47]. Another study examined the antinociceptive and anti-inflammatory mechanisms of *curcumin* in an animal model and reported that *curcumin* may have a central effect and exert its analgesic effect by affecting the opioid system. According to this report, *curcumin* exerts its analgesic effect by enhancing the release and effect of mediators such as dopamine, serotonin, and noradrenaline [44]. *Curcumin* actually increases the number of presynaptic terminals associated with these neurotransmitters and increases their effectiveness by increasing the length of time they are open. *Curcumin* also increases the level of 5-hydroxytryptophan and increases the sensitivity of postsynaptic cells to this substance. It also inhibits monoamine neurotransmitter oxidase A, B enzymes, and increases the level of noradrenaline in the frontal lobe, all of which have analgesic effects [44]. *Curcumin* has also been shown to inhibit inflammatory substances such as cyclooxygenase 2 [47]. It also inhibits pain transmission in the nervous system by affecting L and D phenylalanine isomers. D-alanine is a chemical mediator that suppresses pain, improves general mood, and reduces fatigue and impatience [44]. However, *curcumin*, when administered orally, has little absorption and bioavailability and is rapidly eliminated from the systemic blood flow [26, 32, 44] so that it was poorly measurable in serum even when administered at a daily dose of 12 g [28].. Some researchers have suggested that the skin absorption of *curcumin* is greater than that of the gastrointestinal tract. A study also used a topical ointment containing turmeric to relieve osteoarthritis pain and reported this method as effective [35]. Congruent with the latter study, our results confirm the significant effect of the topical use of *curcumin* on the reduction of knee pain associated osteoarthritis in older adults.

In the present study, while the knee pain reduction in the intervention group continued till the end of the study, the placebo group experienced only a slight reduction in pain at the second time, which did not continue thereafter. The slight reduction of knee pain in the placebo group at the second measurement might partly be attributed to the placebo effect. In other words, assuming that they are using a painkiller, made the patients in this group to express less pain in the second measurement. On the other hand, this group, like the intervention group, massaged the knee while using Vaseline and wrapped it with a cloth. This manner might also have contributed to the reduction of pain in the second measurement. However, because all of these factors were similar for the two groups throughout the study, the persistence of pain reduction in the intervention group can be attributed to the effect of topical application of *curcumin*.

Generally, turmeric is a safe substance and has no reported side effects. Allergies to plants of the ginger family and jaundice are the only restrictions of *curcumin* use. It also makes the skin temporarily yellow. If this discoloration is not important to the patient, *curcumin* ointment can be used as a safe, relatively inexpensive, and effective way to relieve osteoarthritis joint pain.

This study was conducted on a limited sample of elderly patients. Given the scarcity of studies on the effect of topical administration of *curcumin* ointment on joint pain, it is recommended to repeat the same study on larger samples as well as on other age groups with knee joint pain and also for pain relief in other joints. Also, the intervention in the present study continued for 6 weeks. Increasing the duration of treatment and follow-up can clarify whether the analgesic effect can be progressive. In this study, we used a VAS scale to measure knee pain. However, it is suggested that the WOMAC scale be used in future studies. In the present study, we also did not assess the effect of *curcumin* ointment on plasma inflammatory markers. However, it is suggested that some inflammatory markers be evaluated in future studies. Although we tried to keep the patients and the physician blind to the type of the prescribed ointment, however, due to the color of the *curcumin* ointment, the one who trained the patients and assessed the pain was not blind to the type of ointment, which may have affected the pain assessment. It is recommended that a third party assess the pain in future studies. Due to some errors in the values we initially used in the sample size calculation, we recalculated the sample size at the end of the study but the estimated sample size did not change.

## Conclusion

The present study showed that 6 weeks of topical administration of 5% *curcumin* ointment was effective in reducing knee pain in elderly patients with knee osteoarthritis. Therefore, it is recommended to use this ointment in the treatment of elderly patients with knee pain related to osteoarthritis.

## Data Availability

The datasets used and/or analyzed during the current study are available from the corresponding author on reasonable request.

## Abbreviation

T1 – T3: Time 1 - Time 3
